# Molecular Identification and Pathogenicity of *Fusarium* Species Associated with Wood Canker, Root and Basal Rot in Turkish Grapevine Nurseries

**DOI:** 10.3390/jof10070444

**Published:** 2024-06-24

**Authors:** Davut Soner Akgül, Serkan Önder, Nurdan Güngör Savaş, Murat Yıldız, İzzet Bülbül, Mümine Özarslandan

**Affiliations:** 1 Department of Plant Protection, Agriculture Faculty, Çukurova University, 01330 Adana, Türkiye; 2 Department of Plant Protection, Agriculture Faculty, Eskişehir Osmangazi University, 26160 Eskişehir, Türkiye; onderserkan@gmail.com; 3 Manisa Viticulture Research Institute, Turkish Ministry of Agriculture and Forestry, 45125 Manisa, Türkiye; nurdangngrsvs10@gmail.com; 4 Malatya Apricot Research Institute, Turkish Ministry of Agriculture and Forestry, 44090 Malatya, Türkiye; muratyildizbaem@gmail.com; 5 Biological Control Research Institute, Turkish Ministry of Agriculture and Forestry, 01321 Adana, Türkiye; izzetblbl@gmail.com (İ.B.); mumine_deniz@hotmail.com (M.Ö.)

**Keywords:** diversity, *Fusarium annulatum*, *Fusarium curvatum*, *Fusarium nirenbergiae*, phylogenetic analysis, vine decline, Vitis

## Abstract

*Fusarium* species are agriculturally important fungi with a broad host range and can be found as endophytic, pathogenic, or opportunistic parasites in many crop plants. This study aimed to identify *Fusarium* species in bare-rooted, dormant plants in Turkish grapevine nurseries using molecular identification methods and assess their pathogenicity. Asymptomatic dormant plants were sampled from grapevine nurseries (43) in different regions of the country, and fungi were isolated from plant roots and internal basal tissues. The *Fusarium* strains were identified by performing gene sequencing (*TEF1-α*, *RPB2*) and phylogenetic analyses. Pathogenicity tests were carried out by inoculating mycelial agar pieces of strains onto the stem or conidial suspensions into the rhizosphere of vines (1103 Paulsen rootstock). Laboratory tests revealed that *Fusarium* species were highly prevalent in Turkish grapevine nurseries (41 out of 43). Gene sequencing and phylogenetic analyses unraveled that 12 *Fusarium* species (*F. annulatum, F. brachygibbosum, F. clavum, F. curvatum, F. falciforme, F. fredkrugeri, F. glycines, F. nanum, F. nematophilum, F. nirenbergiae, F. solani,* and *Fusarium* spp.) existed in the ready-to-sale plants. Some of these species (*F. annulatum, F. curvatum* and *F. nirenbergiae*) consistently caused wood necrosis of seedling stems, rotting of the basal zone and roots, and reduced root biomass. Although the other nine species also caused some root rot and root reduction, their virulence was not as severe as the pathogenic ones, and they were considered opportunistic parasites or endophytic species. This study suggests that *Fusarium* species might play an important role in root-basal rot, wood canker symptoms, and young vine decline in Turkish grapevine nurseries and that these species need to be considered for healthy seedling production.

## 1. Introduction

Grapevine sapling production is one of the most important agricultural sectors in Türkiye, and many young vines (2.5 to 3 million plants) are produced yearly in different geographical regions in the country [1]. The need for grapevine seedlings in the domestic market is relatively high, and this production needs to be increased to meet the demand for grapevine seedlings in Türkiye.

In grapevine nurseries, abiotic factors (unfavorable weather conditions, nutritional disorders, use of poor-quality production materials, rootstock-scion incompatibility, etc.), nematodes, insects, and fungal pathogens cause the death of plants, and these factors bring about low productivity and economic losses every year. Fungal grapevine trunk disease (GTD) pathogens, which often settle on young seedlings with infected propagation materials, belonging to *Botryosphaeriaceae, Diaporthaceae*, and *Diatrypaceae* families, *Cadophora, Cytospora, Phaeomoniella, Phaeoacremonium Seimatosporium* genera, and soilborne fungi (*Armillaria, Cylindrocarpon*-like anamorphs, *Fusarium, Macrophomina, Phytophthora, Rhizoctonia*, and *Verticillium* sp.) are considered to be the main actors of plant mortality in the nurseries [2,3].

The genus *Fusarium* has an exceptional place in plant pathology, medical mycology, and the food industry as they are both plant and human pathogens and threaten human-animal health by producing mycotoxins in foods. To date, more than 400 *Fusarium* species have been identified, which nested in 23 different species complexes [4]. Most *Fusarium* species are soil-borne and are also called one of the ubiquitous fungal genera in mycology due to their endophytic, saprophytic, hemibiotrophic, or parasitic forms and strong competitive ability. Plant pathogenic species may result in significant crop damage and economic losses in some years by causing root and basal rots, damping-off, seed-tuber-fruit rots, wilt, and head blight diseases. According to the American Phytopathological Society, 83 out of 108 plant species in the field and horticultural crops are affected by one or more *Fusarium* diseases [5]. Many species in the *Fusarium* genus are true plant pathogens, while others are opportunists waiting for soil and environmental conditions to turn unfavorable for plants.

It has been pointed out that root rot-associated fungi considerably reduce young vine health and marketable sapling yield; *Fusarium* and *Cylindrocarpon*-like fungi were the main actors affecting plant vigor, and quality in grapevine nurseries. These fungi cause necrosis in the roots and basal tissues, leading to a reduction in hairy roots, retarded growth, and the death of seedlings or young vines in later stages [6]. Research on the pathogenic roles and diversity of *Fusarium* species on plant death in grapevine nurseries and young vineyards has intensified in recent years. Highet and Nair [7] proved the infection of grapevine hairy roots by *Fusarium oxysporum* through transmission electron microscopy and pathogenicity tests and suggested it would be considered as one of the fungi associated with root rot and decline in the nurseries. Reveglia et al. [8] revealed that the phytotoxins of *Fusarium oxysporum* (such as fusaric acid) and other potential metabolites have a critical role in the occurrence of these symptoms in seedlings and young vines in Australia. Vilvert et al. [9] claimed that *Fusarium oxysporum* f.sp. *herbemontis* is an important species responsible for decline and plant death in Brazilian grapevine nurseries, and it would be possible to control this pathogen using mycorrhizal fungi. Úrbez-Torres et al. [10] stated that *Fusarium* species were common in British Columbia (Canada) vineyards, but the most frequently isolated species might be secondary pathogens on grapevine rootstock 3309C. Similarly, Bustamente et al. [11] suggested that *Fusarium* species isolated from grapevine nurseries and young vineyards in California (USA) are opportunistic pathogens attacking plants under stress. In contrast, Li et al. [12] reported that when the *Fusarium* strains were inoculated into grapevine seedlings, they caused necrosis in the xylem vessels and basal regions of the plants resembling the infections of *Dactylonectria macrodidyma* (a black-foot disease pathogen). Zhang et al. [13] reported for the first time that *Fusarium commune* was a pathogen in grapevines causing leaf yellowing, stunting and root rot in Beijing Region, China. These studies indicate that *Fusarium* species on grapevines are a potential threat to nurseries and newly established vineyards and should not be underestimated. Furthermore, since these species can be found in the latent phase in plants, it is possible to spread them over large areas with marketable grapevine seedlings. Akgül and Ahioğlu [14] detected some *Fusarium* species in young vineyards, along with fungal pathogens associated with grapevine trunk diseases, in southern Türkiye and confirmed the pathogenicity of these species. However, a nationwide study on the diversity and pathogenicity of *Fusarium* species in marketable grapevine saplings is needed. Therefore, this study aimed to identify *Fusarium* species in dormant marketable plants in Turkish grapevine nurseries and to reveal their pathogenicity.

## 2. Materials and Methods

### 2.1. Survey and Isolation of Fusarium Species

The survey was conducted in January 2021 in 43 grapevine nurseries in different geographical regions of Türkiye (in Adıyaman, Bursa, Denizli, Manisa, Mersin, Tekirdağ, Tokat, and Urfa provinces). Ten dormant, commercially ready-for-sale seedlings from each nursery were randomly sampled and transported to the laboratory. The root and basal parts of the seedlings were washed under tap water and disinfected superficially with sodium hypochlorite solution (including >5% active clorine) for 3 min. Root and internal basal tissues (3–4 mm) were placed onto PDA (Potato dextrose agar, CondaLab; Madrid, Spain) containing streptomycin-sulfate (250 mg × L^−1^), and the Petri plates were kept at 25 °C in dark, for ten days to promote fungal colony growth. According to the colony morphology and microscopic characteristics detailed by Leslie and Summerell [15], a single spore was taken from the *Fusarium* colonies and purified on PDA for further stages. Ten Petri plates (containing seven tissue fragments in each) were used, and the isolation frequency of *Fusarium* colonies was calculated by proportioning the tissue number (*Fusarium* detected) to the total number (*n* = 70).

### 2.2. Molecular Identification and Phylogenetic Analyses

Strains were categorized according to colony morphology and microscopic features, and 60 *Fusarium* strains were selected for molecular identification. They were grown on PDA at 25 °C in the dark for seven days, and mycelium (56–60 mg) was harvested for DNA extraction. The genomic DNA was obtained following the CTAB protocol recommended by O’Donnell et al. [16] and diluted with 100 µL PCR grade water (Lonza) and stored at −18 °C for further use. Translation elongation factor (*TEF1-α*) and the second largest protein subunit of RNA polymerase II (*RPB2*) genes were amplified using the primers, EF1/EF2 and RPB2-5f2/fRPB2-7cr, respectively [17]. The PCR reaction mixture contained 5 μL of buffer (10X Green Buffer, DreamTaq Green DNA Polymerase, Thermo-Scientific, Waltham, MA, USA), 2 μL of dNTPs mixture (10 mM each, Thermo Scientific, Waltham, MA, USA), 1 μL of forward and reverse primers (10 pmol·μL^−1^), 0.25 μL of Taq polymerase (DreamTaq Green DNA Polymerase, Thermo-Scientific), 39.75 μL PCR grade water and 1 μL genomic DNA (100 ng·μL^−1^). PCR amplifications were conducted in SimpliAmp A24811™ Thermal Cycler, Applied Biosystems, (Waltham, MA, USA) with the conditions detailed in the publications of O’Donnell et al. [16,17,18]. The PCR products were separated using gel electrophoresis in 1.5% agarose (Invitrogen, Waltham, MA, USA) gel under 55V DC voltage, 250 mA current for 90 min. and were checked for DNA quality visually. After that, PCR products were sequenced bidirectionally via Sanger sequencing, derived chronogram files were trimmed from 3 and 5 prime with CLC main Workbench 5.5, and manual editing was carried out where necessary. Cleaned sequences were compared with those deposited in the NCBI GenBank database using the NCBIblastn suite (National Center for Biotechnology Information). Nucleotide sequences of *TEF1-α* and *RPB2* genes were submitted to the NCBI GenBank, and the accession numbers were obtained. According to the nucleotide BLAST search results of *TEF1-α* and *RPB2*, a representative sequence dataset was used from the NCBI nucleotide database to perform the phylogenetic study. Constructed datasets for *TEF1-α* and *RPB2* sequences were aligned individually via the ClustalW alignment tool in Geneious Prime 2019.1.3 software. After the alignment step, *TEF1-*α and *RPB2* sequences were concatenated from end to end via Geneious Prime 2019.1.3 software for the multi-gene phylogenetic tree. Phylogenetic analyses were based on maximum likelihood (ML). The ML analysis was performed with IQ-TREE on the Galaxy Europe platform [19]. Model Finder was used to determine the best-fit model for the ML tree [20]. ML tree construction was performed under the TIM2e model with equal base frequencies and Invariable + Gamma with four categories (TIM2e + I + G4) in the nucleotide substitution model according to the Bayesian information criterion scores and weights (BIC and w-BIC). For the pseudoreplications of the ML tree, a 1000 ultrafast bootstrap parameter was used [21]. The alignment and the phylogenetic tree were deposited in TreeBASE under the study number S31385 (http://purl.org/phylo/treebase/phylows/study/TB2:S31385, accessed on 23 April 2024).

### 2.3. Pathogenicity Tests

Based on the identification results, 38 *Fusarium* strains were selected for pathogenicity tests, and two types of inoculation methods were followed in order to evaluate from different aspects. In the first, the bark of the dormant cuttings was removed with a sterile cork-borer (3 mm), fresh mycelial agar discs of the strains (10-day old) were placed on these wounds, and these points were wrapped with parafilm™. The cuttings were planted in the pots and grown in greenhouse conditions for four months. The inoculation points were scraped with a scalpel, and necrosis lengths in the wood tissues were measured and recorded [11]. Plants inoculated with an *Ilyonectria liriodendri* (a black-foot disease pathogen) strain (AFP115, obtained from the fungal culture collection of Mycology Lab. in Dept. of Plant Protection in Çukurova University, Adana, Türkiye) were set as positive, and sterile agar-inoculated plants were set as healthy controls. The fungi were re-isolated from the internal tissues at the inoculation points to confirm the pathogenicity by culturing wood chips on a PDA medium. For each seedling, five Petri dishes (seven tissues in each) were used, and the frequency of re-isolation was calculated by proportioning the number of *Fusarium* colonies to the total number of wood chips. In the second trial, rootstock cuttings (cv. 1103 Paulsen) were planted in the plastic pots (0.85 L) containing sterile rooting mix (equal volumes of peat moss and perlite), and the pots were kept in lath house conditions (natural temperature, relative humidity and illumination). The *Fusarium* strains were grown on PDA at 25 °C for 15 days, and conidial suspensions (in sterile distilled water at 10^6^ conidia·ml^−1^ concentration) were prepared using a haemocytometer. Following root formation, conidial suspensions were poured into the root zone of the plants (20 mL per pot), and plants were grown in lath house conditions for four months. The pathogenicity of the strains was assessed based on root dry weight and necrosis length in the plants’ basal zone (in wood tissues). The seedlings were uprooted from the pots, the roots were gently washed under tap water, and were harvested using a pruning shear. After briefly blotting with paper towels, the roots were held in a drying chamber at 65 °C for 48 h, then weighed using a precision balance, and weights were recorded [22]. Nevertheless, the bark of the cuttings was carefully peeled off with a knife, and the length of the necrosis in the wood tissues was measured with a caliper. The *Fusarium* strains were re-isolated from the internal tissues of the basal parts by culturing the tissues on a PDA medium amended with streptomycin-sulfate (250 mg·L^−1^), and the re-isolation frequency was calculated as mentioned above. Pathogenicity tests were arranged according to the design of the randomized plot with six replications (two plants in a replicate), and twelve plants were used for each strain. The trials were repeated once (2022 and 2023 years), and the data were subjected to statistical analysis. To clarify the virulence of each *Fusarium* species, an analysis of variance (ANOVA) was performed again on the mean values of all strains belonging to the same species.

### 2.4. Statistical Analyses

ANOVA was performed on lengths of wood necrosis in basal parts and internodes of the stems and root dry weights. The data were checked for normality, and root square transformation was applied. Means were compared using Fisher’s least significant difference (LSD) test at the 5% significance level [23].

## 3. Results

### 3.1. Fungal Isolation and Prevalence of Fusarium Species

The first *Fusarium* colonies were aroused on the internal basal tissues and hairy roots of marketable, dormant grapevine plants after 5–6 days of incubation (at 24 °C in the dark) in PDA media. Through colony morphology (colony reverse color, appearance of mycelia), 779 *Fusarium* colonies were detected in 3010 plant tissues (in 430 Petri dishes) plated for 43 grapevine nurseries. Their colony morphology is shown in Supplementary Materials. The isolation frequency of *Fusarium* species in these nurseries is shown in Table 1.

As shown in Table 1, *Fusarium* species were detected in 41 out of 43 grapevine nurseries, and the prevalence of these species in Turkish grapevine nurseries was calculated at 95.3%. The isolation frequency in nurseries ranged between 2.9 and 65.7%, while the overall average was 24.9%. Nevertheless, black foot, Petri disease pathogens, *Botryosphaeriaceae fungi*, *Cytospora*, *Diaporthe*, *Truncatella* species, and soil-borne plant pathogenic fungi (*Macrophomina* and *Rhizoctonia*) were also found. Considering different geographical regions and morphological-microscopic characteristics, 121 *Fusarium* colonies were pre-selected for molecular-phylogenetic analyses.

### 3.2. Molecular Identification and Phylogenetic Analyses

**Figure 1:**
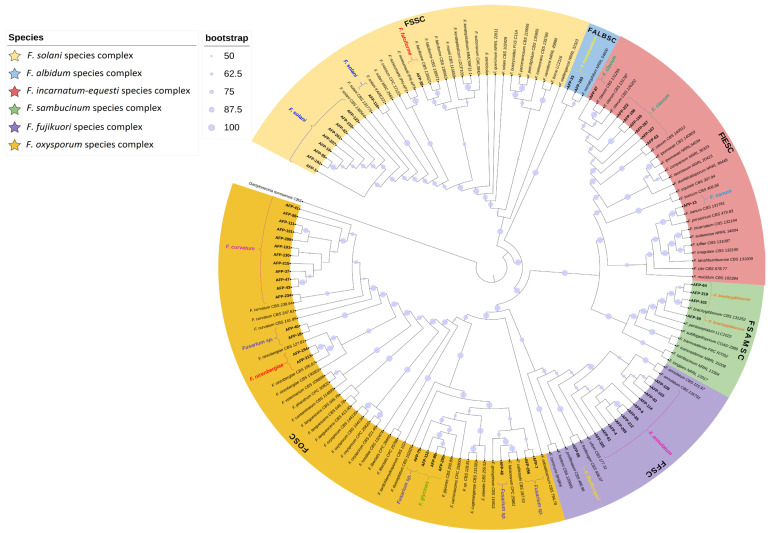
Multi gene (*TEF1-α* and *RPB2*) Maximum Likelihood tree of *Fusarium* strains. Circles of different sizes show bootstrap support values from 1000 replicates which are indicated at the nodes. Bootstrap values less than 50% are not shown. The bold characters represent the Turkish strains. *Dactylonectria torresensis* CBS 129086 was used for rooting the ML tree.

Using the primers EF1/EF2 and RPB2-5f2/fRPB2-7cr, TEF1-α and RPB2 gene regions of the *Fusarium* strains were amplified by conventional PCR, and agarose gel electrophoresis revealed DNA bands with sizes ranging from 680 to 1600 bp (respectively). The initial approach for the identification of strains relied on a blastn search of partial sequences of the *TEF1-α* and *RPB2* gene. NCBIblastn search was performed with the nucleotide sequences of these regions, and the strains were 99.2–100% similar to other *Fusarium* species in the GenBank. Afterwards, these sequences were aligned with the closest matching and nearly closest references obtained from GenBank (Table 2) for resolve the ambiguities. However, the NCBIblastn search results from the two gene regions of all strains were not parallel, and the second gene region in some strains matched with different *Fusarium* species. Yet, phylogenetic analyses conducted with concatenated nucleotide sequences clarified the ambiguity observed in the blastn results. To construct the phylogenetic tree, we used a dataset comprising 150 taxa, including 60 local strains, 89 reference *Fusarium* strains, and one strain from the *Dactylonectria* genus. The dataset contained 3040 nucleotide sites. The phylogenetic tree was rooted using the *Dactylonectria torresensis* strain CBS 129086. In the aligned final dataset, out of the 3040 nucleotide sites, 1787 were identified as constant, and 1001 were identified as parsimony informative. Additionally, 1598 nucleotide sites were found to have distinct site patterns. Model finder analysis of the aligned dataset indicated that the TIM2e + I + G4 model was the most appropriate for constructing the maximum likelihood (ML) tree, with a BIC score of 43,876.5755 and a w-BIC score of 0.785. The constructed ML tree showed a sum of branch lengths of 2.5465 and a sum of internal branch lengths of 1.2670 (in Figure 1).

In this study, 60 *Fusarium* strains (Table 3) included in the phylogenetic analyses were clustered in six different species complexes, of which 38.3% were *F. oxysporum* (FOSC), 20% *F. fujikuroi* (FFSC), 18.3% *F. solani* (FSSC), 13.3% *F. incarnatum-equiseti* (FIESC), 6.7% *F. sambucinum* (FSAMSC) and 3.3% *F. albidum*. The *Fusarium* species clustered into these species complexes distributed in 12 species (Figure 1): *F. annulatum* (11 strains—18.3%), *F. brachygibbosum* (4 strains—6.7%), *F. clavum* (7 strains—11.6%), *F. curvatum* (12 strains—20%), *F. falciforme* (one strain—1.6%), *F. fredkrugeri* (one strain—1.6%), *F. glycines* (3 strains—5.0%), *F. nanum* (one strain—1.6%), *F. nematophilum* (2 strains—3.3%), *F. nirenbergiae* (2 strains—3.3%), *F. solani* (10 strains—16.7%), and *Fusarium* sp. (6 strains—10.0%).

### 3.3. Pathogenicity of Fusarium Strains and Species

**Figure 2:**
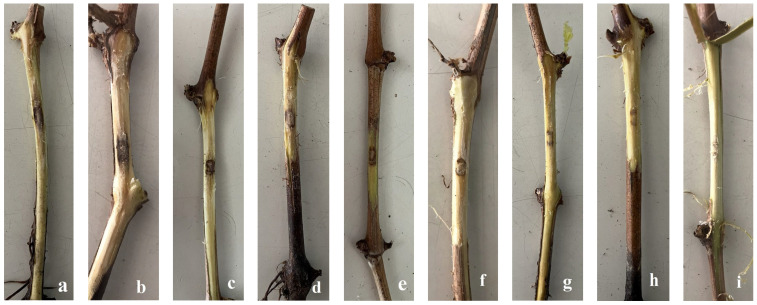
Wood necrosis in the stems of grapevine seedlings induced by *Fusarium* and *Ilynonectria liriodendri* after four months of inoculation. (**a**) *I. liriodendri*, (**b**) *F. annulatum*, (**c**) *F. brachygibbosum*, (**d**) *F. nirenbergiae*, (**e**) *F. curvatum*, (**f**) *F. solani*, (**g**) *F. glycines*, (**h**) *F. falciforme*, and (**i**) non-inoculated control.

In four-month pathogenicity tests, some Fusarium strains inoculated on the plants’ stems (considering the possibility of contamination to the vines through pruning wounds) produced lesions ranging from 5.9 to 12.0 mm (Figure 2). The lesion lengths of 10 of the 38 strains inoculated on the plants in the first year were longer than those of the control and other strains and statistically different.

The majority of these strains belonged to the following species: *F. annulatum* (seven strains), *F. brachygibbosum* (one strain), *F. nirenbergiae* (one strain), and *I. liriodendri*. In the second year, only nine strains had lesions longer than other *Fusarium* strains and control statistically. They were *F. annulatum* (five strains), *F. nirenbergiae* (two strains), *F. curvatum* (one strain) and *I. liriodendri* (Table 4). In both years, lesions caused by other *Fusarium* strains were not significantly longer than those of the control. However, these strains could be re-isolated from the point of inoculation (except from the non-inoculated control) at rates ranging from 15.2% to 53.8%.

**Figure 3:**
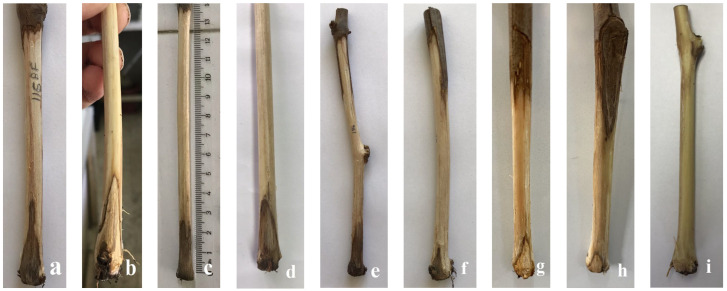
Basal wood necrosis in grapevine seedlings induced by *Fusarium* and *Ilynonectria liriodendri* after four months of inoculation. (**a**) *I. liriodendri*, (**b**) *F. annulatum*, (**c**) *F*. *nirenbergiae*, (**d**) F. curvatum, (**e**) *F. glycines*, (**f**) *F. solani*, (**g**) *F. fredkrugeri*, (**h**) *F. falciforme*, and (**i**) non-inoculated control.

In the second type of pathogenicity test, 38 strains increased basal rot and significantly reduced root dry weight compared to the non- inoculated control. Due to the large number of strains and replicates, there was a large variance among the means of strains, resulting in many statistical groups. The *Fusarium* strains, and *I. liriodenri* caused basal rot of the wood tissues in the basal region of the seedlings (Figure 3), and their lengths ranged from 3.6 to 37.0 mm in the first year and from 3.7 to 7.8 mm in the second year (Table 5). As in the case of stem necrosis, *F. annulatum*, *F. curvatum*, and *I. liriodendri* species were found to cause the most extended necrose length in basal rot formation. However, the wood necrosis induced by the other species was not as severe and consistent as that of these three species. The *Fusarium* strains and *I. liriodendri* could be re-isolated from the basal necroses (except from the non-inoculated control) at rates ranging from 10.3% to 36.8%.

In parallel to basal rot, the *Fusarium* strains and *I. liriodendri* decreased hair root formation and root dry weight in the inoculated plants compared with the non-inoculated control plants. In 30–35% of the plants inoculated with *F. annulatum*, *F. curvatum*, and *I. liriodendri*, shoots dried up, and plants died after one month. The average root dry weight recorded per plant in the first year varied between 0.022 and 0.344 g, while in the second year, these values were recorded between 0.599 and 1.463 g (Table 6).

**Figure 4:**
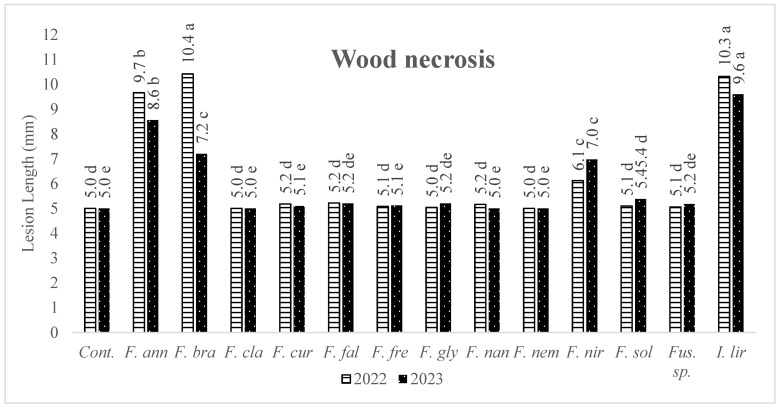
The average lesion lengths in the wood tissues of grapevine seedlings (1103 Paulsen rootstock) induced by *Fusarium* species and *I. liriodendri*. Cont: Control, *F. ann*: *Fusarium annulatum*, *F. bra*: *Fusarium brachygibbosum*, *F. cla*: *Fusarium clavum*, *F. cur*: *Fusarium curvatum*, *F. fal*: *Fusarium falciforme*, *F. fre*: *Fusarium fredkrugeri*, *F. gly*: *Fusarium glycines*, *F. nan*: *Fusarium nanum*, *F. nem*: *Fusarium nematophilum*, *F. nir*: *Fusarium nirenbergiae*, *F. sol*: *Fusarium solani*, and *I. lir*: *Ilyonectria liriodendri*. Means accompanied by same letter are not significantly different (*p* = 0.05) according to LSD tests.

**Figure 5:**
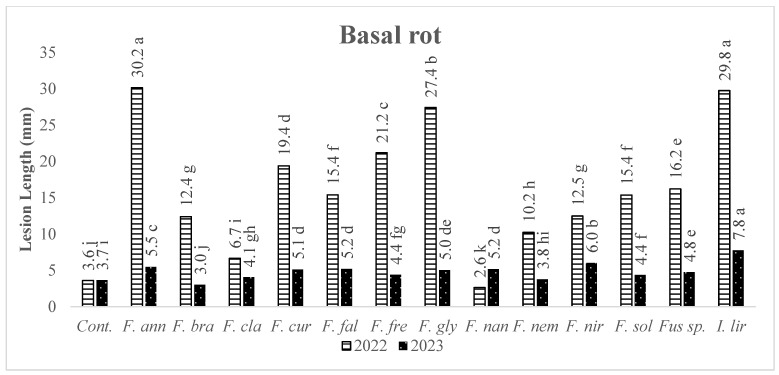
The average lesion lengths in the basal wood tissues of grapevine seedlings (1103 Paulsen rootstock) induced by *Fusarium* species and *I. liriodendra*. Cont: Control, *F. ann*: *Fusarium annulatum*, *F. bra*: *Fusarium brachygibbosum*, *F. cla*: *Fusarium clavum*, *F. cur*: *Fusarium curvatum*, *F. fal*: *Fusarium falciforme*, *F. fre*: *Fusarium fredkrugeri*, *F. gly*: *Fusarium glycines*, *F. nan*: *Fusarium nanum*, *F. nem*: *Fusarium nematophilum*, *F. nir*: *Fusarium nirenbergiae*, *F. sol*: *Fusarium solani*, and *I. lir*: *Ilyonectria liriodendri*. Means accompanied by same letter are not significantly different (*p* = 0.05) according to LSD tests.

The average values (necrosed lengths in stem and basal part, and dry root weights) of the strains (belonging to the same species) were considered, and ANOVA was performed on the means to clarify the pathogenicity of each *Fusarium* species. When the pathogenicity of *Fusarium* species was evaluated according to the length of necrosis at the inoculation point, it was found that *F. annulatum*, *F. brachygibbosum*, *F. nirenbergiae* and *Ilyonectria liriodendri* caused wood necrosis. In contrast, the others did not show the same influence (Figure 4). Regarding the effects of *Fusarium* species on basal rot formation, it was determined that the three species causing the most extended necrosis in the first year were *F. annulatum*, *I. liriodendri*, and *F. glycines*; in the second year, *I. liriodendri*, *F. nirenbergiae*, and *F. annulatum*, respectively (Figure 5).

**Figure 6:**
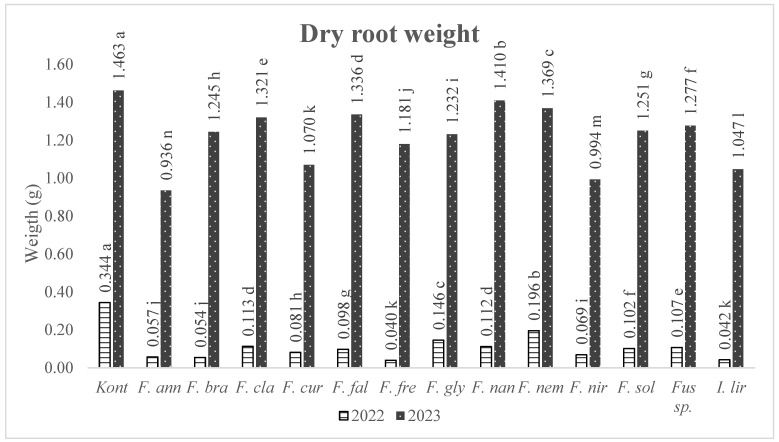
The average dry root weight of grapevine seedlings (1103 Paulsen rootstock) induced by *Fusarium* species and *I. liriodendri*. Cont: Control, *F. ann*: *Fusarium annulatum*, *F. bra*: *Fusarium brachygibbosum*, *F. cla*: *Fusarium clavum*, *F. cur*: *Fusarium curvatum*, *F. fal*: *Fusarium falciforme*, *F. fre*: *Fusarium fredkrugeri*, *F. gly*: *Fusarium glycines*, *F. nan*: *Fusarium nanum*, *F. nem*: *Fusarium nematophilum*, *F. nir*: *Fusarium nirenbergiae*, *F. sol*: *Fusarium solani*, and *I. lir*: *Ilyonectria liriodendri*. Means accompanied by same letter are not significantly different (*p* = 0.05) according to LSD tests.

The effect of *Fusarium* species on root dry weight reduction was almost parallel to basal rot; when the results of both years were generally evaluated, *F. annulatum, I. liriodendri*, and *F. nirenbergiae* were found to be the most effective species (Figure 6).

## 4. Discussion

In this study, *Fusarium* fungi were found to be relatively common (95.3% of the nurseries) in bare-rooted plants ready for sale in Turkish grapevine nurseries. This rate is considerably higher than that found in North America and Canada, but it is close to that in the nurseries in France and Spain. Garnett et al. [24] investigated the fungal species associated with root rot of grapevines in two different vineyards in California and isolated a high proportion of *Fusarium* species (together with *Rhizoctonia*, *Pythium*, *Macrophomina*, *Phytophthora* fungi) in the sampled vines. Torres et al. [10] found *Fusarium* species in 43.9% of the seedlings ready for sale in four grapevine nurseries in Canada and determined that these species were isolated from the plants between 20.0 and 86.7%. Bustamente et al. [11] determined that the incidence of *Fusarium* species was 36.7% in young vineyards and 31.7% in nursery plants in California. However, Pintos et al., [25] detected 92% to 98% of *Fusarium* fungi, among other GTD pathogens, from plants sampled from two commercial grapevine nurseries in Spain and one in France.

In the current study, 12 distinct *Fusarium* species were found in six *Fusarium* species complexes in the grapevine nurseries, with the most common species complexes being *F. oxysporum* (38.3%), *F. fujikuroi* (20.0%), and *F. solani* (18.3%). The results revealed more diversity of *Fusarium* species than previous studies conducted in Canada and United States. Urbez-Torres et al. [10] reported that *Fusarium* species diversity was very low in four nurseries in British Columbia (Canada) and found only two species from two different species complexes (*F*. *oxysporum* and *F. fujikuroi*). However, Bustamente et al. [11] reported a high diversity of *Fusarium* in young vines in California (total, nine *Fusarium* species in six species complexes) and found five species (*F. annulatum, Fusarium* sp., *F. solani, F. keratoplasticum, F. nirenbergiae*) belonging to these complexes in the nurseries. The fungal isolation results in our study, the high *Fusarium* species diversity in ready-to-sale grapevine seedlings, and the presence of joint species *(F. annulatum, F. brachygibbosum, F. clavum, F. nirenbergiae, F. solani*) in the plants were consistent with the findings of Bustamente et al. [11]. However, in the abovementioned studies, *F. avenaceum, F. ramigenum, F. culmorum, F. keratoplasticum, F. oxysporum*, and *F. proliferatum* were not found in grapevine nurseries in Türkiye. Interestingly, although *F. oxysporum* is a large species complex, including 21 species [26], and *F. oxysporum* has an essential place in this complex, we could not detect *F. oxysporum* among the *Fusarium* species we isolated from vines. In contrast to our findings, Zeidan et al. [27] reported that *F. oxysporum* strains obtained from different grape varieties (cv. Crimson Seedless, Flame, King Robi, Superior, and Thompson), showing wilt and root rot in Egypt were pathogenic by producing polygalacturonase and cellulase enzymes. These strains were associated with wilt in Crimson Seedless but with root rot in other cultivars. When phylogenetic analyses were performed, it was revealed that many strains similar to this species were *F. curvatum, F. glycines*, and *F. nirenbergiae*. Similarly, although *F. proliferatum* has been reported as an important root rot pathogen in maize, soybean, tomato, and grapevine [10,28,29,30], we could not detect *F. proliferatum* among the available grapevine *Fusarium* strains. These differences may have been made possible by detailed phylogenetic analyses using concatenated genes such as *TEF1-α* and *RPB2*, which are highly recommended to identify *Fusarium*. O’Donnell et al. [4] suggested that when identifying *Fusarium* species, the *TEF1α* and *RPB2* gene regions should be amplified and concatenated to perform phylogenetic analyses if possible, and in case of financial limitations, the sequence of the *TEF1α* region might be sufficient.

Based on the pathogenicity results of *Fusarium* strains inoculated on grapevine stems, *F. annulatum, F. brachygibbosum, F. curvatum*, and *F. nirenbergiae* were found to produce more considerable wood necrosis in comparison to the control and other *Fusarium* species. Reveglia et al. [7] widely isolated *F. oxysporum* strains from grapevines showing young vine decline symptoms (in Italy) and investigated their phytotoxic metabolites. The fusaric acid purified from these strains caused severe necrosis when injected into tobacco leaves, and they suggested that this metabolite may also cause root and basal rot in grapevines. Akgül and Ahioğlu [14] inoculated *F. brachygibbosum* strains (obtained from three-year-old young grapevines) on the stems of grapevine seedlings and determined it to be a highly virulent species in woody tissues. Rajput et al. [31] investigated the pathogenicity of *F. equiseti* strains isolated from the trunks of grapevines in the Kunar province of Afghanistan and reported that tissue necrosis occurred when this species was inoculated on woody shoots of three-year-old plants. Bustamente et al. [11] inoculated *F. annulatum, F. nirenbergiae*, and *F. solani* strains from young grapevines on the stems of one-year-old vines and revealed that after seven months; these species produced longer necroses in the wood tissues of plants when compared to non-inoculated controls. The results of these studies support the view that the wounds occurring via disbudding of cuttings or basal cuts in the seedlings or wounds by removing vine suckers on trunks (in the vineyards) may be susceptible to *Fusarium* infections and that *Fusarium* species may be involved in young vine decline or trunk diseases.

Another outcome from the pathogenicity tests was that some *Fusarium* species (*F. annulatum, F. curvatum, F. nirenbergiae, F. solani*) significantly increased root-basal rot and reduced root biomass in the inoculated plants. Highet and Nair [7] investigated the effect of *Fusarium oxysporum* infections on root rot development in grapevines (cv. Semillon 5–25 years old) in New Zealand. When plants were inoculated with *F. oxysporum*, they observed the disintegration of bark cells (by transmission electron microscopy) and determined that *Fusarium*-infected root cells lacked cytoplasm compared to uninfected cells. Vilvert et al. [9] stated that *F. oxysporum* f.sp. *herbemontis* was an important fungal pathogen in Brazilian grapevine nurseries, causing basal rot, reduction in root biomass, and root rot symptoms in infected vines. Zhang et al. [13] detected several *Fusarium* species from young grapevines (cv. Red Globe) showing decline and leaf yellowing in vineyards in Beijing, China and found that *F. commune* was pathogenic among these species and associated with these symptoms. Li et al. [12] revealed that *Fusarium* strains inoculated on grapevine seedlings caused not only a reduction in root biomass, root rot but also interveinal discolorations and coalescent necrosis on the leaves of the plant. When these strains were inoculated together with *Dactylonectria macrodidyma*, the severity of the disease was further increased.

Regarding the pathogenicity of other *Fusarium* species, *F. annulatum* has also been reported to cause fruit-corm and root rot in crop plants such as melon and onion, as well as medicinal-aromatic plants such as *Blettila striata* L. and saffron, in addition to vine [32,33,34,35]. These studies indicate that *F. annulatum* is pathogenic in many hosts. *F. nirenbergiae* and *F. curvatum* were other virulent species in the pathogenicity tests on the grapevine seedlings. When we reviewed the studies on this subject, we found only one study [11] in which *F. nirenbergiae* was detected as a pathogen in grapevine. However, in other studies, it has been reported to be pathogenic in crop plants such as maize, passion fruit, almond (in Portugal and Spain), and maple. Sanna et al. [29] investigated *Fusarium* species associated with post-emergence damping-off and root rot in maize and found that *F. nirenbergiae* caused a disease index of over 50% in some maize areas of Italy, as did *F. verticilloides, F. annulatum* and *F. commune*. Aiello et al. [36] identified *F. nirenbergiae* as the cause of root rot and wilt in passion fruit plants. Zhao et al. [37] identified it as the cause of wilt in maple trees (in China), and Moral-Lopez et al., [38] in almonds (in Portugal and Spain). Another virulent species, *F. curvatum*, which we identified in pathogenicity tests, has previously been found to be associated with dieback disease in *Dendrobium officinale* [39] and leaf spot of cherry [40] in China.

In this study, although other *Fusarium* species (*F. clavum, F. falciforme, F. fredkrugeri, F. glycines, F. nanum, F. nematophilum, F. solani*) from Turkish grapevine nurseries reduced root biomass in grapevine seedlings compared to the control, their virulence was not as consistent as *F. annulatum, F. brachygibbosum, F. curvatum*, and *F. nirenbergiae*. These results support the view that most of the *Fusarium* species may be present in grapevine seedlings as parasites in the current study. However, it has been determined that these species with low virulence here were highly virulent in other crop plants, causing root and fruit rots in these plants and thus resulting in yield losses. Medeiros-Araújo et al. [41] characterized *Fusarium* species (*F. falciforme, F. kalimantanense, F. pernambucanum,* and *F. sulawesiense*) associated with fruit rot of muskmelon in Brazil. From these species, *F. falciforme* and *F. sulawesiense* were found to be more aggressive than others. Zhang et al. [42] detected that

*F. nanum* strains obtained from muskmelon fields in China were also associated with fruit rot. In another study conducted in Vietnam, *F. falciforme* was found to be an aggressive species causing wilt in chrysanthemum [43]. *F. clavum* was reported as a plant pathogen causing leaf spot and fruit rot on tomato, and petal brown spot on rose in Italy [44,45]. These studies indicate that different *Fusarium* species are pathogenic in different plants, while they have no significant effect in others. It is conceivable that host–pathogen interactions may be important in these events. Environmental factors (temperature, precipitation, relative humidity, soil structure-microbiome, etc.), the physiology and biochemistry of host plants, and gene contents of *Fusarium* fungi are also suggested to influence the availability of metabolites and toxins necessary for fungal growth and pathogenicity, which in turn influence the host–pathogen interaction [46,47]. Some studies also suggest that *Fusarium* species provide various benefits to plants by enhancing plant growth, triggering production of secondary metabolites, and protection against pathogens [48]. Jelenic et al. [49] found endophytic *F. solani* and *F. subglutinans* to reduce gray mold incidence (caused by *Botrytis cinerea*) on grape clusters, increase yield, and provide plants with more robust development under unfavorable weather conditions. However, no detailed biochemical or transcriptomics explanation underlying these positive effects could be suggested. Such studies reveal that much remains unknown about the interactions of *Fusarium* species in plants and their function in agriculture in phytopathological aspects.

## 5. Conclusions

This study revealed that grapevine nurseries in Türkiye are rich in *Fusarium* species, most of which are pathogenic and associated with root-basal rot and wood necrosis. Phylogenetic analysis and detailed molecular identification tests (sequencing at least two descriptive gene regions for the *Fusarium* genus) have become inevitable since it is impossible to distinguish between visually identical species. In pathogenicity tests, it has been suggested that not only root dry weight, but also basal rot and wood cancer necrosis should be taken into account to identify prominent virulent species (as in *F. annulatum, F. curvatum*, and *F. nirenbergiae*). The rhizosphere of plants contains diverse microbial communities, such as actinomycetes, bacteria, fungi, and protozoa, which interact with plant roots and each other. Climatic conditions, soil texture, chemistry, and plants’ genetic assets may closely influence the formation of these communities and plant–pathogen interactions in the rhizosphere [50,51]. These factors may affect the resistance of plants to pathogens and may also play a role in the transition of *Fusarium* species, which were found to be weakly parasitic in this study, to the pathogenic form. Pathogenic *Fusarium* species should be considered in grapevine nurseries, and various biological and chemical control possibilities should be investigated in the future.

## Figures and Tables

**Table 1:** Location of surveyed grapevine nurseries, rootstock/cultivars, and isolation frequency of Fusarium species in Türkiye.

Nursery	Location	Rootstock or Cultivar	Isolation
			Frequency (%)
1	Bursa	1103P—Trakya İlkeren	32.9
2	Mersin	1103P—Victoria	32.9
3	Salihli, Manisa	Thompson Seedless	-
4	Salihli, Manisa	Sultana Seedless	28.5
5	Salihli, Manisa	Sultana Seedless	-
6	Salihli, Manisa	Sultana Seedless	38.5
7	Salihli, Manisa	1103P/Sultana Seedless	40.0
8	Alaşehir, Manisa	Sultana Seedless	34.3
9	Alaşehir, Manisa	Sultana Seedless	4.3
10	Alaşehir, Manisa	Sultana Seedless	24.3
11	Sarıgöl, Manisa	Sultana Seedless	58.6
12	Salihli, Manisa	Sultana Seedless	17.1
13	Tekirdağ	Kober 5BB/Sultan 1	8.6
14	Tekirdağ	Kober 5BB/Bozbey	17.2
15	Tekirdağ	1103P—Tekirdağ Çekirdeksizi	27.1
16	Tekirdağ	110R-Yapıncak	2.9
17	Denizli	41B/Sultana Seedless	20.0
18	Denizli	41B/Sultana Seedless	40.0
19	Denizli	41B/Sultana Seedless	65.7
20	Denizli	41B/Sultana Seedless	50.0
21	Denizli	41B/Michele Palieri	45.7
22	Şanlıurfa	1103P—Ergin Çekirdeksizi	12.9
23	Şanlıurfa	110R—Horozkarası	11.5
24	Şanlıurfa	99R—Çiloreş	12.9
25	Şanlıurfa	1103P—Victoria	5.7
26	Manisa	41B/Red Globe	47.1
27	Manisa	Kober 5BB/Royal	20.0
28	Manisa	1103P—Sultana Seedless	20.0
29	Manisa	Kober 5BB—Sultana Seedless	21.4
30	Manisa	1103P—Crimson Seedless	17.1
31	Manisa	110R/Alicante Bouschet	26.3
32	Alaşehir, Manisa	1103P—Thompson Seedless	60.0
33	Manisa	Kober 5BB/Ata Sarısı	22.9
34	Turgutlu, Manisa	Kober 5BB/Sultana Seedless	28.6
35	Manisa	Kober 5BB/Trakya İlkeren	8.6
36	Tokat	1103P—Narince	40.0
37	Tokat	1103P/Narince	21.4
38	Tokat	1103P/Narince	11.4
39	Tokat	1103P/Sultan7	12.9
40	Tokat	1103P/Narince	20.0
41	Tokat	Du Lot/Narince	12.9
42	Adıyaman	Kober 5BB/Hatun Parmağı	17.9
43	Mersin	1103P/Victoria	31.3
Mean			24.9

**Table 2:** GenBank accession numbers of partial sequence of *TEF1-α* and *RPB2* of references species used in the phylogenetic analyses.

Reference Species	Strains	Location (Country: City)	GenBank Accession Numbers	
			*TEF1-α*	*RPB2*
*F. solani*	CBS 138564	Türkiye	KT272100	KT272102
“	CBS 131775	Iran	JX118990	JX237778
“	KARE_221	USA: California	MK077042	MK077080
“	MRC_2565	N/A	MH582420	MH582410
“	CBS 102429	Australia	KM231936	KM232376
*F. crassum*	CPC_37122	South Africa	MW248760	MW446594
*F. noneumartii*	IPN-AP1	Mexico	OP902594	OP902591
“	IPN-AP3	Mexico	OP902596	OP902593
*F. falciforme*	CBS 135521	Mexico	KU711733	KU604357
“	CBS 138971	N/A	KT716212	KT716187
“	CBS 138963	N/A	KT716213	KT716188
*F. martii*	CBS 115659	Germany	JX435156	JX435256
*F. keratoplasticum*	LDCF109	China	OP184958	OP186372
“	MMC59F11-1	N/A	MF069182	MF069181
*F. suttonianum*	CML3942	N/A	MK158921	MH709236
*F. stericicola*	N/A	N/A	LR583659	LR583888
*F. quercinum*	NRRL:22611	USA: Michigan	DQ246841	EU329518
*F. bostrycoides*	FUS C11A	Italy	PP105767	PP125181
*F. parceramosum*	CBS 115695	South Africa	JX435149	JX435249
*F. petroliphilum*	CBS 135955	Türkiye	KU711768	KU604337
*F. metavorans*	CBS 135789	Greece	KU711773	KU604374
*F. vanettenii*	NRRL 45880	N/A	FJ240352	JX171655
*F. breve*	LC2116	China: Ganzhou	MW620163	MW474688
*F. waltergamsii*	NRRL 32323	USA: Pennsylvania	DQ246951	EU329576
*F. nematophilum*	NRRL_54600	N/A	N/A	JX171664
*F. clavum*	CBS 131255	Iran	MN170460	MN170393
“	CBS 131787	Iran	MN170461	MN170394
“	CBS 126202	Namibia	MN170456	MN170389
“	CBS 140912	Russia	MN170462	MN170395
*F. ipomoeae*	CBS 140909	Russia	MN170479	MN170412
“	NRRL 34034	USA	GQ505636	GQ505814
*F. compactum*	NRRL 36323	USA	GQ505648	GQ505826
*F. lacertarum*	NRRL 20423	USA	GQ505593	GQ505771
*F. duofalcatisporum*	NRRL 36448	USA	GQ505652	GQ505830
*F. equiseti*	CBS 307.94	Germany	KR071777	KU604327
*F. toxicum*	CBS 406.86	Germany	MN170508	MN170441
*F. nanum*	CBS 131781	Iran	MN170487	MN170420
*F. persicinum*	CBS 479.83	N/A	MN170495	MN170428
*F. incarnatum*	CBS 132194	Thailand	KF255470	KF255542
*F. sulawense*	NRRL 34004	USA	GQ505628	GQ505806
*F. luffae*	CBS 131097	Iran	MN170482	MN170415
*F. irregulare*	CBS 132190	Thailand	MN170480	MN170413
*F. tanahbumbuense*	CBS 131009	Iran	MN170506	MN170439
*F. citri*	CBS 678.77	Japan	MN170453	MN170386
*F. mucidum*	CBS 102394	El Salvador	MN170484	MN170417
*F. brachygibbosum*	CBS 131252	Iran	JQ429334	JX162526
*F. pentaseptatum*	LLC1022	Ethiopia	OP487255	OP486819
*F. subflagellisporum*	COAD 2989	Brazil	MT774486	MZ970426
*F. transvaalense*	FRC R7052	N/A	MW233161	MW233505
“	NRRL 31008	Australia	MW233102	MW233446
*F. sambucinum*	NRRL 13394	USA: Pennsylvania	MW233064	MW233407
*F. longipes*	NRRL 13317	USA: California	MW233058	MG282411
*F. annulatum*	CBS 115.97	Italy	MW401973	MW402785
“	CBS 135791	Greece	MW402054	MW402746
*F. udum*	CBS 177.31	South Africa	MH484957	MH484866
*F. fredkrugeri*	CBS 408.97	USA: Maryland	MW402126	MW402814
*F. proliferatum*	CBS 480.96	Papua New Guinea	MN534059	MN534272
*F. foetens*	CBS 120665	Iran	MH485009	MH484918
*F. commune*	BHBR5	N/A	OR900978	OR888540
*F. odoratissimum*	CBS 794.70	Iran	MH484969	MH484878
*F. callistephi*	CBS 187.53	Netherlands	MH484966	MH484875
*F. fabacearum*	CPC 25801	South Africa	MH485029	MH484938
*F. gossypinum*	CBS 116611	Cote d’Ivoire	MH484998	MH484907
*F. elaeidis*	CBS 255.52	N/A	MH484965	MH484874
*F. cugenangense*	CBS 131393	Australia	MH485019	MH484928
*Fusarium sp.*	CBS 128.81	USA	MH484975	MH484884
*F. carminascens*	CPC 25800	South Africa	MH485028	MH484937
*F. glycines*	CBS 200.89	Argentina	MH484979	MH484888
*F. duoseptatum*	CBS 102026	Malaysia	MH484987	MH484896
*F. tardichlamydosporum*	CBS 102028	Malaysia	MH484988	MH484897
*F. libertatis*	CPC 25788	South Africa	MH485024	MH484933
“	CPC 28465	South Africa	MH485035	MH484944
*F. hoodiae*	CBS 132474	South Africa	MH485020	MH484929
*F. oxysporum*	CBS 221.49	South East Asia	MH484963	MH484872
“	CPC 25822	South Africa	MH485034	MH484943
“	CBS 144134	Germany	MH485044	MH484953
“	CBS 144135	Germany	MH485045	MH484954
*F. languescens*	CBS 413.90	Israel	MH484981	MH484890
“	CBS 646.78	Morocco	MH484972	MH484881
“	CBS 645.78	Morocco	MH484971	MH484880
*F. contaminatum*	CBS 114899	Germany	MH484992	MH484901
*F. pharetrum*	CPC 30822	South Africa	MH485042	MH484951
*F. veterinarium*	CBS 109898	Netherlands	MH484990	MH484899
*F. nirenbergiae*	CBS 130301	USA	MH485017	MH484926
“	CBS 196.87	Italy	MH484977	MH484886
“	CBS 127.81	USA	MH484974	MH484883
*F. curvatum*	CBS 141.95	Netherlands	MH484985	MH484894
“	CBS 247.61	Germany	MH484967	MH484876
“	CBS 238.94	Netherlands	MH484984	MH484893
*Dactylonectria torresensis*	CBS 129086	Portugal: Torres Vedras	JF735870	KM232347

N/A: Data not available.

**Table 3:** Location of surveyed grapevine nurseries, cultivars, and the species found with their *TEF1-α* and *RPB2* gene sequence numbers.

Strains	Species	Location	Rootstock/Cultivar	GenBank Accession Numbers	
				*TEF1-α*	*RPB2*
AFP004	*Fusarium annulatum*	Bursa	1103 Paulsen	PP449277	PP449217
AFP006	″	Bursa	1103 Paulsen	PP449278	PP449218
AFP061	″	Manisa	Kober 5BB	PP449279	PP449219
AFP082	″	Tokat	1103 Paulsen	PP449280	PP449220
AFP085	″	Tokat	1103 Paulsen	PP449281	PP449221
AFP103	″	Manisa	Kober 5BB	PP449282	PP449222
AFP109	″	Manisa	110 Richter	PP449283	PP449223
AFP114	″	Manisa	Kober 5BB	PP449284	PP449224
AFP212	″	Tokat	1103 Paulsen	PP449285	PP449225
AFP265	″	Manisa	Sultana Seedless	PP449286	PP449226
AFP320	″	Tekirdağ	1103 Paulsen	PP449287	PP449227
AFP059	*Fusarium brachygibbosum*	Manisa	Kober 5BB	PP449288	PP449228
AFP064	″	Manisa	41B	PP449289	PP449229
AFP102	″	Manisa	Ramsey	PP449290	PP449230
AFP219	″	Şanlıurfa	1103 Paulsen	PP449291	PP449231
AFP062	*Fusarium clavum*	Manisa	Kober 5BB	PP449292	PP449232
AFP087	″	Tokat	1103 Paulsen	PP449293	PP449233
AFP107	″	Manisa	Ramsey	PP449294	PP449234
AFP150	″	Tokat	1103 Paulsen	PP449295	PP449235
AFP196	″	Manisa	Ramsey	PP449296	PP449236
AFP222	″	Tokat	1103 Paulsen	PP449297	PP449237
AFP267	″	Manisa	Sultana Seedless	PP449298	PP449238
AFP037	*Fusarium curvatum*	Denizli	140 Ruggeri	PP449299	PP449239
AFP041	″	Denizli	1103 Paulsen	PP449300	PP449240
AFP043	″	Denizli	140 Ruggeri	PP449301	PP449241
AFP047	″	Denizli	140 Ruggeri	PP449302	PP449242
AFP096	″	Manisa	Kober 5BB	PP449303	PP449243
AFP101	″	Manisa	Ramsey	PP449304	PP449244
AFP111	″	Manisa	110 Richter	PP449305	PP449245
AFP130	″	Mersin	1103 Paulsen	PP449306	PP449246
AFP191	″	Tokat	1103 Paulsen	PP449307	PP449247
AFP208	″	Tokat	1103 Paulsen	PP449308	PP449248
AFP215	″	Şanlıurfa	1103 Paulsen	PP449309	PP449249
AFP234	″	Şanlıurfa	1104 Paulsen	PP449310	PP449250
AFP038	*Fusarium falciforme*	Denizli	140 Ruggeri	PP449311	PP449251
AFP066	*Fusarium fredkrugeri*	Manisa	Kober 5BB	PP449312	PP449252
AFP098	*Fusarium glycines*	Manisa	Ramsey	PP449313	PP449253
AFP112	″	Manisa	110 Richter	PP449314	PP449254
AFP295	″	Manisa	Sultana Seedless	PP449315	PP449255
AFP013	*Fusarium nanum*	Mersin	140 Ruggeri	PP449316	PP449256
AFP033	*Fusarium nematophilum*	Manisa	Kober 5BB	PP449317	PP449257
AFP163	″	Tokat	1103 Paulsen	PP449318	PP449258
AFP194	*Fusarium nirenbergiae*	Manisa	Ramsey	PP449319	PP449259
AFP213	″	Tokat	1103 Paulsen	PP449320	PP449260
AFP001	*Fusarium solani*	Bursa	1103 Paulsen	PP449321	PP449261
AFP019	″	Manisa	1103 Paulsen	PP449322	PP449262
AFP042	″	Denizli	140 Ruggeri	PP449323	PP449263
AFP095	″	Manisa	Kober 5BB	PP449324	PP449264
AFP116	″	Manisa	Kober 5BB	PP449325	PP449265
AFP123	″	Mersin	1103 Paulsen	PP449326	PP449266
AFP153	″	Tokat	1103 Paulsen	PP449327	PP449267
AFP192	″	Manisa	Ramsey	PP449328	PP449268
AFP207	″	Mersin	1103 Paulsen	PP449329	PP449269
AFP261	″	Manisa	Sultana Seedless	PP449330	PP449270
AFP007	*Fusarium* sp.	Bursa	1103 Paulsen	PP449331	PP449271
AFP018	″	Manisa	1103 Paulsen	PP449332	PP449272
AFP040	″	Denizli	140 Ruggeri	PP449333	PP449273
AFP048	″	Denizli	1103 Paulsen	PP449334	PP449274
AFP075	″	Denizli	140 Ruggeri	PP449335	PP449275
AFP256	″	Manisa	Sultana Seedless	PP449336	PP449276

AFP (Asma *Fusarium* Projesi in Turkish).

**Table 4:** Mean wood lesion lengths caused by *Fusarium* species in the inoculation points of 1103 Paulsen rootstock plants after four months.

Strains	2022	Necrosis		Strains	2023	Necrosis	
	Species	(mm)			Species	(mm)	
AFP006	*F. annulatum*	12.0	a *	AFP061	*F. annulatum*	8.9	a *
AFP114	*F. annulatum*	11.9	a	AFP115	*Ilyonectria liriodendri*	8.6	a
AFP109	*F. annulatum*	11.0	b	AFP213	*F. nirenbergiae*	8.4	a
AFP103	*F. annulatum*	10.9	bc	AFP103	*F. annulatum*	8.0	b
AFP059	*F. brachygibbosum*	10.4	bc	AFP265	*F. annulatum*	7.7	bc
AFP115	*Ilyonectria liriodendri*	10.3	c	AFP114	*F. annulatum*	7.5	c
AFP265	*F. annulatum*	7.9	d	AFP111	*F. curvatum*	7.5	c
AFP194	*F. nirenbergiae*	7.1	e	AFP194	*F. nirenbergiae*	7.5	c
AFP061	*F. annulatum*	6.9	e	AFP006	*F. annulatum*	6.3	d
AFP004	*F. annulatum*	6.9	e	AFP004	*F. annulatum*	5.9	de
AFP096	*F. curvatum*	5.3	f	AFP096	*F. curvatum*	5.2	e
AFP213	*F. nirenbergiae*	5.3	f	AFP109	*F. annulatum*	5.1	e
AFP256	*Fusarium* sp.	5.3	f	AFP059	*F. brachygibbosum*	5.1	e
AFP043	*F. curvatum*	5.2	f	AFP043	*F. curvatum*	5.1	e
AFP101	*F. curvatum*	5.2	f	AFP101	*F. curvatum*	5.0	e
AFP038	*F. falciforme*	5.2	f	AFP098	*F. glycines*	5.0	e
AFP040	*Fusarium* sp.	5.2	f	AFP019	*F. solani*	5.0	e
AFP013	*F. nanum*	5.2	f	AFP041	*F. curvatum*	5.0	e
AFP130	*F. curvatum*	5.1	f	AFP130	*F. curvatum*	5.0	e
AFP019	*F. solani*	5.1	f	AFP037	*F. curvatum*	5.0	e
AFP037	*F. curvatum*	5.1	f	AFP040	*Fusarium* sp.	5.0	e
AFP075	*Fusarium* sp.	5.1	f	AFP066	*F. fredkrugeri*	5.0	e
AFP095	*F. solani*	5.1	f	AFP123	*F. solani*	5.0	e
AFP111	*F. curvatum*	5.1	f	AFP256	*Fusarium* sp.	5.0	e
AFP191	*F. curvatum*	5.1	f	AFP191	*F. curvatum*	5.0	e
AFP041	*F. curvatum*	5.1	f	AFP038	*F. falciforme*	5.0	e
AFP001	*F. solani*	5.1	f	AFP001	*F. solani*	5.0	e
AFP007	*Fusarium* sp.	5.1	f	AFP018	*Fusarium* sp.	5.0	e
AFP222	*F. clavum*	5.1	f	AFP222	*F. clavum*	5.0	e
AFP261	*F. solani*	5.1	f	AFP261	*F. solani*	5.0	e
AFP066	*F. fredkrugeri*	5.1	f	AFP075	*Fusarium* sp.	5.0	e
AFP033	*F. nematophilum*	5.0	f	AFP033	*F. nematophilum*	5.0	e
AFP048	*Fusarium* sp.	5.0	f	AFP048	*Fusarium* sp.	5.0	e
AFP062	*F. clavum*	5.0	f	AFP062	*F. clavum*	5.0	e
AFP123	*F. solani*	5.0	f	AFP095	*F. solani*	5.0	e
AFP196	*F. clavum*	5.0	f	AFP196	*F. clavum*	5.0	e
AFP098	*F. glycines*	5.0	f	AFP013	*F. nanum*	5.0	e
AFP018	*Fusarium* sp.	5.0	f	AFP007	*Fusarium* sp.	5.0	e
Non-inoculated Control		5.0	f	Non-inoculated Control		5.0	e

AFP (Asma *Fusarium* Projesi in Turkish). * Means accompanied by same letter are not significantly different (*p* = 0.05) according to LSD tests.

**Table 5:** Mean basal necrose lengths caused by *Fusarium* species in 1103 Paulsen rootstock plants after four months.

Strains	2022	Basal		Strains	2023	Basal	
	Species	Necrosis (mm)			Species	Necrosis (mm)	
AFP061	*F. annulatum*	37.0	a	AFP115	*Ilyonectria liriodendri*	7.8	a *
AFP103	*F. annulatum*	34.6	ab	AFP004	*F. annulatum*	6.2	ab
AFP114	*F. annulatum*	30.8	a–c	AFP101	*F. curvatum*	6.2	ab
AFP041	*F. curvatum*	29.8	a–d	AFP194	*F. nirenbergiae*	6.2	ab
AFP115	*Ilyonectria liriodendri*	29.8	a–d	AFP103	*F. annulatum*	6.0	a–c
AFP004	*F. annulatum*	28.4	a–e	AFP111	*F. curvatum*	6.0	a–c
AFP109	*F. annulatum*	27.6	b–f	AFP114	*F. annulatum*	5.8	b–d
AFP098	*F. glycines*	27.4	b–f	AFP213	*F. nirenbergiae*	5.8	b–d
AFP006	*F. annulatum*	26.8	b–g	AFP037	*F. curvatum*	5.6	b–e
AFP265	*F. annulatum*	26.4	b–g	AFP109	*F. annulatum*	5.6	b–e
AFP019	*F. solani*	25.2	c–h	AFP256	*Fusarium* sp.	5.4	b–f
AFP111	*F. curvatum*	23.8	c–i	AFP006	*F. annulatum*	5.2	b–f
AFP018	*Fusarium* sp.	23.2	c–i	AFP013	*F. nanum*	5.2	b–f
AFP256	*Fusarium* sp.	22.8	c–i	AFP038	*F. falciforme*	5.2	b–f
AFP037	*F. curvatum*	21.2	d–j	AFP048	*Fusarium* sp.	5.2	b–f
AFP066	*F. fredkrugeri*	21.2	d–j	AFP061	*F. annulatum*	5.2	b–f
AFP075	*Fusarium* sp.	19.8	d–j	AFP191	*F. curvatum*	5.2	b–f
AFP096	*F. curvatum*	19.0	f–k	AFP040	*Fusarium* sp.	5.0	b–f
AFP123	*F. solani*	17.8	g–l	AFP098	*F. glycines*	5.0	b–f
AFP101	*F. curvatum*	17.0	h–m	AFP265	*F. annulatum*	5.0	b–f
AFP043	*F. curvatum*	16.0	i–m	AFP075	*Fusarium* sp.	4.8	b–g
AFP007	*Fusarium* sp.	15.4	i–n	AFP123	*F. solani*	4.8	b–g
AFP038	*F. falciforme*	15.4	i–n	AFP001	*F. solani*	4.6	b–g
AFP130	*F. curvatum*	15.0	i–n	AFP019	*F. solani*	4.6	b–g
AFP261	*F. solani*	15.0	i–n	AFP041	*F. curvatum*	4.6	b–g
AFP191	*F. curvatum*	13.6	j–o	AFP043	*F. curvatum*	4.6	b–g
AFP194	*F. nirenbergiae*	12.8	j–o	AFP222	*F. clavum*	4.6	b–g
AFP059	*F. brachygibbosum*	12.4	j–p	AFP007	*Fusarium* sp.	4.4	b–g
AFP213	*F. nirenbergiae*	12.2	j–p	AFP066	*F. fredkrugeri*	4.4	b–g
AFP095	*F. solani*	10.4	k–q	AFP096	*F. curvatum*	4.4	b–g
AFP033	*F. nematophilum*	10.2	k–q	AFP130	*F. curvatum*	4.2	c–g
AFP040	*Fusarium* sp.	9.8	l–q	AFP018	*Fusarium* sp.	4.0	d–g
AFP001	*F. solani*	8.6	m–q	AFP095	*F. solani*	4.0	d–g
AFP196	*F. clavum*	8.4	m–q	AFP261	*F. solani*	4.0	d–g
AFP062	*F. clavum*	6.6	n–q	AFP033	*F. nematophilum*	3.8	e–g
AFP048	*Fusarium* sp.	6.4	n–q	AFP062	*F. clavum*	3.8	e–g
AFP222	*F. clavum*	5.2	o–q	AFP196	*F. clavum*	3.8	e–g
AFP013	*F. nanum*	3.6	p–q	AFP059	*F. brachygibbosum*	3.7	f–g
Non-inoculated Control		2.6	q	Non-inoculated Control		3.0	g

AFP (Asma *Fusarium* Projesi in Turkish). * Means accompanied by same letter are not significantly different (*p* = 0.05) according to LSD tests.

**Table 6:** Mean root dry weights of 1103 Paulsen rootstock plants inoculated with *Fusarium* species after four months.

Strains	2022	Root Dry		Strains	2023	Root Dry	
	Species	Weight (g)			Species	Weight (g)	
Non-inoculated Control		0.344	a	Non-inoculated Control		1.463	a *
AFP041	*F. curvatum*	0.224	b	AFP222	*F. clavum*	1.436	b
AFP033	*F. nematophilum*	0.196	c	AFP048	*Fusarium* sp.	1.425	c
AFP256	*Fusarium* sp.	0.182	d	AFP062	*F. clavum*	1.417	d
AFP123	*F. solani*	0.150	e	AFP013	*F. nanum*	1.410	e
AFP098	*F. glycines*	0.146	f	AFP101	*F. curvatum*	1.399	f
AFP018	*Fusarium* sp.	0.134	g	AFP033	*F. nematophilum*	1.369	g
AFP062	*F. clavum*	0.125	h	AFP075	*Fusarium* sp.	1.353	h
AFP261	*F. solani*	0.125	h	AFP261	*F. solani*	1.349	i
AFP048	*Fusarium* sp.	0.119	i	AFP038	*F. falciforme*	1.336	j
AFP013	*F. nanum*	0.112	j	AFP001	*F. solani*	1.327	k
AFP130	*F. curvatum*	0.111	j	AFP095	*F. solani*	1.286	l
AFP196	*F. clavum*	0.109	jk	AFP007	*Fusarium* sp.	1.282	m
AFP019	*F. solani*	0.108	k	AFP019	*F. solani*	1.266	n
AFP109	*F. annulatum*	0.104	l	AFP040	*Fusarium* sp.	1.251	o
AFP222	*F. clavum*	0.103	l	AFP059	*F. brachygibbosum*	1.245	p
AFP038	*F. falciforme*	0.098	m	AFP098	*F. glycines*	1.232	q
AFP004	*F. annulatum*	0.087	n	AFP256	*Fusarium* sp.	1.231	q
AFP001	*F. solani*	0.083	o	AFP066	*F. fredkrugeri*	1.181	r
AFP040	*Fusarium* sp.	0.082	o	AFP191	*F. curvatum*	1.172	s
AFP007	*Fusarium* sp.	0.078	p	AFP103	*F. annulatum*	1.160	t
AFP194	*F. nirenbergiae*	0.072	q	AFP018	*Fusarium* sp.	1.118	u
AFP096	*F. curvatum*	0.070	q	AFP196	*F. clavum*	1.108	v
AFP213	*F. nirenbergiae*	0.065	r	AFP096	*F. curvatum*	1.101	w
AFP043	*F. curvatum*	0.063	r	AFP006	*F. annulatum*	1.098	x
AFP114	*F. annulatum*	0.058	s	AFP213	*F. nirenbergiae*	1.069	y
AFP059	*F. brachygibbosum*	0.054	t	AFP043	*F. curvatum*	1.056	z
AFP101	*F. curvatum*	0.054	t	AFP115	*Ilyonectria liriodendri*	1.047	a1
AFP103	*F. annulatum*	0.051	tu	AFP123	*F. solani*	1.026	b1
AFP006	*F. annulatum*	0.050	u	AFP111	*F. curvatum*	1.016	c1
AFP075	*Fusarium* sp.	0.049	uv	AFP109	*F. annulatum*	1.012	d1
AFP095	*F. solani*	0.047	vw	AFP037	*F. curvatum*	0.974	e1
AFP037	*F. curvatum*	0.045	wx	AFP041	*F. curvatum*	0.921	f1
AFP111	*F. curvatum*	0.044	x	AFP130	*F. curvatum*	0.919	f1
AFP115	*Ilyonectria liriodendri*	0.042	xy	AFP194	*F. nirenbergiae*	0.919	f1
AFP066	*F. fredkrugeri*	0.040	yz	AFP114	*F. annulatum*	0.901	g1
AFP191	*F. curvatum*	0.038	z	AFP004	*F. annulatum*	0.893	h1
AFP265	*F. annulatum*	0.026	a1	AFP265	*F. annulatum*	0.880	i1
AFP061	*F. annulatum*	0.022	b1	AFP061	*F. annulatum*	0.599	j1

AFP (Asma *Fusarium* Projesi in Turkish) * Means accompanied by same letter are not significantly different (*p* = 0.05) according to LSD tests.

## Data Availability

The original contributions presented in the study are included in the article, further inquiries can be directed to the corresponding author.

## Supplementary Materials

The following supporting information can be downloaded at: https://www.mdpi.com/article/10.3390/jof10070444/s1, Figure S1: Colony morphology of Fusarium species identified in this study (on PDA, incubated at 25 °C, for 21 days); (a) *F. annulatum*, (b) *F. brachygibbosum*, (c) *F. clavum*, (d) *F. curvatum*, (e) *F. falciforme*, (f) *F. fredkrugeri*, (g) *F. glycines*, (h) *F. nanum*, (i) *F. nematophilum*, (j) *F. nirenbergiae*, (k) *F. solani*, (l) *Fusarium* sp.
