# Supplementary material for: Molecular Identification and Pathogenicity of Fusarium Species Associated with Wood Canker, Root and Basal Rot in Turkish Grapevine Nurseries
Source: J Fungi (Basel). 2024 Jun 24;10(7):444. doi: 10.3390/jof10070444 (PMC11278196; doi:10.3390/jof10070444)
Supplement: Supplementary file 1 [file jof-10-00444-s001.zip › jof-3038454-supplementary.pdf]

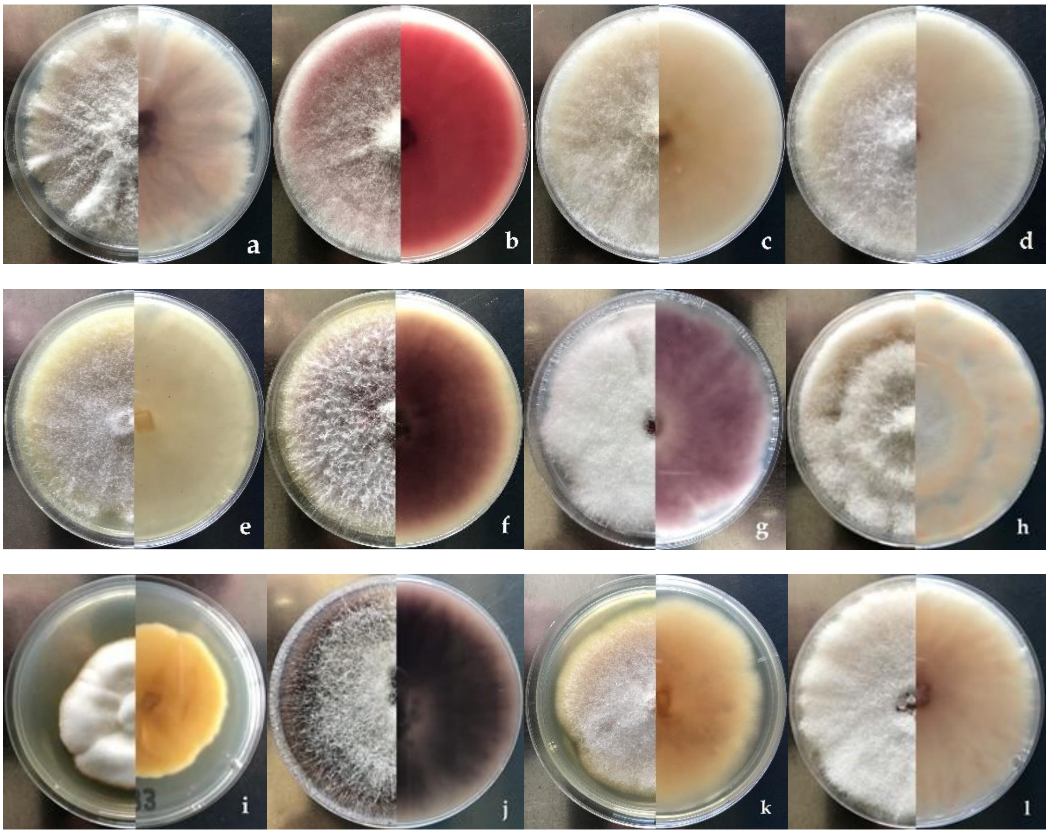

Figure S1. Colony morphology of *Fusarium* species identified in this study (on PDA, incubated at 25°C, for 21 days); a) *F. annulatum*, b) *F. brachygibbosum*, c) *F. clavum*, d) *F. curvatum*, e) *F. falciforme*, f) *F. fredkrugeri*, g) *F. glycines*, h) *F. nanum*, i) *F. nematophilum*, j) *F. nirenbergiae*, k) *F. solani*, l) *Fusarium* sp.
